# Comparative Genome Analysis and Spore Heat Resistance Assay Reveal a New Component to Population Structure and Genome Epidemiology Within *Clostridium perfringens* Enterotoxin-Carrying Isolates

**DOI:** 10.3389/fmicb.2021.717176

**Published:** 2021-09-08

**Authors:** Kaisa Jaakkola, Kira Virtanen, Päivi Lahti, Riikka Keto-Timonen, Miia Lindström, Hannu Korkeala

**Affiliations:** ^1^Department of Food Hygiene and Environmental Health, University of Helsinki, Helsinki, Finland; ^2^Department of Bacteriology and Immunology, Human Microbiome Research Program, Faculty of Medicine, University of Helsinki, Helsinki, Finland; ^3^Northern Finland Laboratory Centre NordLab, Oulu, Finland; ^4^City of Helsinki, Unit of Environmental Services, Helsinki, Finland

**Keywords:** *Clostridium perfringens*, core genome, enterotoxin (CPE), spore heat resistance, gastrointestinal disease, comparative genome analysis

## Abstract

*Clostridium perfringens* causes a variety of human and animal enteric diseases including food poisoning, antibiotic-associated diarrhea, and necrotic enteritis. Yet, the reservoirs of enteropathogenic enterotoxin-producing strains remain unknown. We conducted a genomic comparison of 290 strains and a heat resistance phenotyping of 30 *C. perfringens* strains to elucidate the population structure and ecology of this pathogen. *C. perfringens* genomes shared a conserved genetic backbone with more than half of the genes of an average genome conserved in >95% of strains. The c*pe*-carrying isolates were found to share genetic context: the *cpe*-carrying plasmids had different distribution patterns within the genetic lineages and the estimated pan genome of *cpe*-carrying isolates had a larger core genome and a smaller accessory genome compared to that of 290 strains. We characterize *cpe*-negative strains related to chromosomal *cpe-*carrying strains elucidating the origin of these strains and disclose two distinct groups of chromosomal *cpe*-carrying strains with different virulence characteristics, spore heat resistance properties, and, presumably, ecological niche. Finally, an antibiotic-associated diarrhea isolate carrying two copies of the enterotoxin *cpe* gene and the associated genetic lineage with the potential for the emergence of similar strains are outlined. With *C. perfringens* as an example, implications of input genome quality for pan genome analysis are discussed. Our study furthers the understanding of genome epidemiology and population structure of enteropathogenic *C. perfringens* and brings new insight into this important pathogen and its reservoirs.

## Introduction

*Clostridium perfringens* is an anaerobic, spore former causing gas gangrene, wound infections, and a variety of human and animal diseases involving the gastrointestinal (GI) system (Kiu and Hall, 2018). Based on the recent estimation, this pathogen originated 40–80 000 years ago, and strains similar to the current isolates have been isolated from a 12 000-year-old mummified puppy and a 5,000-year-old mummified human corpse (Lugli et al., 2017; Feng et al., 2020). Presently, *C. perfringens* is a common human enteric pathogen: approximately 15% of reported antibiotic-associated diarrhea (AAD) cases are caused by *C. perfringens* (Asha et al., 2006), and the amount of *C. perfringens* food-poisoning cases in the European Union has been projected to be between 850 000 and 5 million cases per year (Silva et al., 2015).

Genome sequencing and comparative analysis have shown that *C. perfringens* has a large accessory genome reflecting the wide variety of caused diseases and produced toxins (Petit et al., 1999; Rood et al., 2018). Recently, five phylogenetic lineages (I–V) of *C. perfringens* have been identified (Kiu et al., 2017; Feng et al., 2020; Abdel-Glil et al., 2021). There is currently no consensus on naming these lineages, and different nomenclature has been used in previous studies. Feng et al. (2020) estimated that the core genome (genes present in 95% of genomes) shared by these five lineages comprised only one-third of the full genome of each lineage, emphasizing the size and diversity of this species’ accessory genomes.

Identified genetic lineages are not yet established in use for the classification of *C. perfringens* strains. Instead, five toxins are used to toxinotype strains to types A–G (Rood et al., 2018). Strains of different toxinotypes are associated with different host preferences and cause different diseases: for example, toxinotype B strains are associated with sheep dysentery and toxinotype G strains carrying NetB toxin with necrotic enteritis in chickens (Rood et al., 2018). Both food-poisoning and AAD cases of *C. perfringens* diarrhea are primarily caused by type F strains (previously *cpe*-carrying type A strains), which produce pore-forming *C. perfringens* enterotoxin (CPE) but not β-toxin, ε-toxin, or ι-toxin (Rood et al., 2018). The sole chromosomal gene in the toxinotyping scheme is alpha (*plc*), present in all *C. perfringens* strains, while the other toxinotyping toxins are carried on transposable elements or a family of conjugative plasmids (Li et al., 2013). Toxinotyping, therefore, does not reflect the phylogenetic lineage of strains. Recently, a virulence gene profile scheme (I–XV), including chromosomal genes for typing of foodborne isolates (Abdelrahim et al., 2019), has been proposed.

The current understanding is that all *C. perfringens* types can carry the *cpe* gene and produce CPE, but that only approximately 5% of the strains are *cpe*-positive (Miyamoto et al., 2006). The 319 aa *cpe* gene is located either in pCPF5603 or pCPF4969 plasmid (plasmid-mediated *cpe*, p-cpe strains) or on a transposable element Tn*5565* integrated into the chromosome (chromosomal *cpe*, c-cpe strains) (Cornillot et al., 1995; Brynestad et al., 1997; Miyamoto et al., 2006; Li et al., 2010). Foodborne outbreaks are caused by both p-cpe and c-cpe strains (Lahti et al., 2008). Additionally, a 325 aa variant of the *cpe* gene of unknown clinical relevance has been described in the pCPBB1 plasmid (Miyamoto et al., 2011). Known *cpe*-carrying plasmids are all conjugative and horizontally transferable (Miyamoto et al., 2006).

The current typing practices do not reveal the genetic lineage, and likewise, the source of enteric *C. perfringens* infection is often not identified. Transmission routes and ecology of *C. perfringens* are poorly understood, and the reservoirs of enteropathogenic strains remain unknown. *C. perfringens* is known as an environmental bacteria associated with soil, water, sewage, and dust, but also the GI tract of humans and animals (Hatheway, 1990). Suggested reservoirs for *cpe*-positive strains include healthy animals and humans, sludge, soil, and retail meat (Wen and McClane, 2004; Heikinheimo et al., 2006; Li et al., 2007; Lindström et al., 2011; Li et al., 2013; Lahti et al., 2012; Hu et al., 2018). C-cpe strains have been solely isolated from food items or food poisoning associated samples, while p-cpe strains are often associated with non-food-borne human disease and animal enteric disease (Collie and McClane, 1998; Sparks et al., 2001). Comparative genome analysis results have suggested that c-cpe strains are clonal (Deguchi et al., 2009; Xiao et al., 2012) and specialized to yet unknown environmental niche. The GI tract has been suggested to be the adapted niche for p-cpe strains (Lahti et al., 2012). Particularly, pCPF5603 plasmid-carrying strains are associated with care home outbreaks and AAD (Miyamoto et al., 2006; Kiu et al., 2019). A recent study on human enteric isolates suggested that *C. perfringens* strains from a single source might also persist and cause longitudinal outbreaks spanning several years (Kiu et al., 2019), supporting the hypothesis of healthy humans serving as reservoirs for *cpe*-carrying strains (Heikinheimo et al., 2006).

Some *C. perfringens* strains produce heat-resistant spores which enable them to proliferate in foods after cooking if the cold chain is compromised (Sarker et al., 2000; Li and McClane, 2006b; Lindström et al., 2011). The heat resistance of *C. perfringens* spores has been studied widely (Weiss and Strong, 1967; Ando et al., 1985; Sarker et al., 2000; Raju and Sarker, 2005; Li and McClane, 2006a, b, 2008; Xiao et al., 2015), and the main finding is that c-cpe strains produce heat-resistant spores with the D_99°C_ value average of 53.2 min (Weiss and Strong, 1967; Sarker et al., 2000). The known key mechanism for this spore heat resistance phenotype is the ability of strains to produce a variant of small, acid-soluble protein (Ssp4), which binds strongly to the spore DNA to protect it (Li et al., 2009). Contrarily, p-cpe strains and cpe negative strains produce heat-sensitive spores with low D_99°C_ values (on average 1.0 min) (Sarker et al., 2000). The link between genotype and phenotype has not been studied previously, and only three strains with known D values have been sequenced. The growth temperatures of different strains have not been widely studied.

To elucidate the ecology and reservoirs of *cpe*-carrying *C. perfringens* strains, we determined the heat resistance phenotypes and growth temperatures for 30 sequenced *C. perfringens* strains. To understand the population structure and genetic context of this pathogen, the pangenome, cgMLST typing, and phylogenetic tree of 290 *C. perfringens* strains were created. Our results suggest that the presence of the *cpe* gene is associated with certain genetic backgrounds instead of just horizontally transferable enterotoxin. We also describe two distinct groups of chromosomal *cpe*-carrying strains with different virulence characteristics, spore heat resistance properties, and, presumably, ecological niche. The importance of input genome quality for pangenome analysis is discussed as a larger core genome, and a smaller pangenome of *C. perfringens* compared to recent estimations is presented.

## Materials and Methods

### Bacterial Strains and Genomes

A total of 30 *C. perfringens* strains isolated from various sources during 1984–2007 were sequenced and used for heat resistance and growth temperature assays (Supplementary Table 1). The strains included food poisoning associated isolates from foods (*n* = 8) or feces (*n* = 6); human cases of antibiotic-associated diarrhea (*n* = 3); feces of healthy people (*n* = 8); and feces of healthy production animals (*n* = 2), soil (*n* = 1), and sludge (*n* = 2). The strains, their isolation source, and type of *cpe* locus have been previously reported (Heikinheimo et al., 2006; Lahti et al., 2008, 2012). Twenty-seven isolates carried the *cpe* gene, and three were cpe-negative. The *cpe* gene was present in the chromosome of 11 strains (c-cpe) and a plasmid in 16 strains (p-cpe). The *cpe* genetic location on p-cpe strains was either on pCPF4969/IS1470-like (*n* = 9) or pCPF5603/IS1151 (n = 8) plasmid (Heikinheimo et al., 2006; Lahti et al., 2008 2012).

The *cpe*-negative *C. perfringens* strains ATCC 13124 and str. 13, and c-cpe strain SM101 were used as reference genomes (Shimizu et al., 2002; Myers et al., 2006). For phylogenetic and pangenome analyses also publicly available *C. perfringens* genomes (n = 260), representing all described genetic lineages, and including 56 *cpe-*carrying isolates, were included (Supplementary Table 2). Genomes were downloaded from the Patric database (Gillespie et al., 2011).

### Genome Sequencing and Annotation

Genomic DNA of 30 *C. perfringens* strains was extracted (Keto-Timonen et al., 2006), and whole-genome sequencing was performed using PacBio RSII (Institute of Biotechnology, Helsinki, Finland). Sequenced genomes were assembled using HGAP3 and checked for circularity using Gap4 (Staden et al., 2003; Chin et al., 2013). To improve the draft assembly, Illumina MiSeq reads and Pilon tool were used for genome polishing (Walker et al., 2014). Sequenced *C. perfringens* genomes were deposited in the GenBank (accession numbers listed in Supplementary Table 3). Both sequenced and downloaded genomes were annotated using Prokka (Seemann, 2014). Protein functional annotation and conserved domain predictions were refined using CD-search and CDD (Li and Godzik, 2006; Marchler-Bauer et al., 2009).

### cgMLST

To determine the cgMLST target gene set and to create a genome-wide gene-by-gene comparison, ChewBBACA was used (Silva et al., 2018). Both custom-made schema and schema created by Abdel-Glil (2019) available at https://www.cgmlst.org (accessed 10.01.2021) were used. The custom-made schema was created using closed *C. perfringens* genome ATCC 13124 as a training genome and selected genomes as initial query (Supplementary Table 1). Orthologous coding sequences (CDSs) were called using blast score ratio equal to or greater than 0.6 as the threshold. Paralogs, genes shorter than 200 bp, and genes without start or stop codons were excluded, and only the longest allele was kept in the list. Listed genes were used to call alleles from query genomes (Supplementary Table 2). In both called schemes, a threshold of 95 was selected for further analysis (9 genomes removed). Called alleles were once again pruned for paralogs, and the cgMLST gene list was created by filtering out alleles present in less than 95% of genomes.

### Pangenome and Comparative Genome Analyses

For the core genome and pangenome analysis, Roary (Page et al., 2015) and Panaroo (Tonkin-Hill et al., 2020) pipelines were used. The genomes were submitted to the Roary pangenome pipeline v3.6.1 using parameters to split paralogs and 95% minimum percentage identity for blastp. The Panaroo pipeline was run in strict mode and using the default parameter to split paralogs. Similar parameters were used for the creation of pangenomes for 86 *cpe*-carrying strains and 63 lineage IV strains. The core genome clusters were functionally annotated with Eggnog 5.0 (Huerta-Cepas et al., 2019). Scoary was used to associate gene clusters with observed phenotypes (Brynildsrud et al., 2016).

Initially, the Roary pipeline produced a noticeably small core genome, and to produce comparable results with the Panaroo pipeline, a set of quality criteria for input genomes was applied. Genomes were required to meet two or more quality indicators to be included in pangenome analysis (Parrello et al., 2019). Used quality criteria were (1) Patric Genome Quality coarse consistency over 97.0%; (2) Patric Genome Quality fine consistency over 92.0%; (3) less than 100 alleles over 20% shorter than reference sequence in Abdel-Glil cgMLST scheme (Supplementary Tables 2, 10). In total, seven genomes (BER-NE33, W1319, PBS5, PBD1, PC5, K473, and T3381) were excluded from the Roary analysis of 283 strains based on these criteria.

Additional genome comparisons were performed using bi-directional BLASTP with the standard scoring matrix BLOSUM62 and an initial E-value cut-off of 1e^–05^. Two genes were acknowledged orthologs if a reciprocal best blast hit existed among them, and both hits had over 90% similarity and 80% coverage. On average identified bi-directional best hits between any two strains shared over 95% identity (data not shown). When manual curation revealed putative orthologs with conserved synteny but sequence identity below 80%, a term divergent ortholog is used. Genomes were aligned and visually compared with progressive Mauve (Darling et al., 2010) and SEED Viewer 2.0 (Overbeek et al., 2014). Comparison tools available at Patric database (Gillespie et al., 2011; Wattam et al., 2016) were also used for manual curation.

### Phylogenetic Analysis

Maximum likelihood phylogenetic trees were constructed using fasttree 2 (Price et al., 2010). Minimal spanning tree was calculated with Grapetree (Zhou et al., 2018) using MSTreeV3. Phylogenetic trees based on Roary calculated core genomes and cgMLST were visualized and annotated with Grapetree and iTOL v3^1^.

### Heat-Resistance Assay for Spores and Vegetative Cells

The heat-resistance phenotype and estimated D values of 30 *C. perfringens* spores and vegetative cells were determined according to established procedures (Weiss and Strong, 1967; Ando et al., 1985; Sarker et al., 2000). All strains were prepared as described by Duncan and Strong (1968) and Ando et al. (1985). Briefly, Duncan-Strong (DS) medium cultures grown for 24 h at 37°C were heat-shocked at 75°C for 15 min to kill the vegetative cells and facilitate spore germination. A 100 μl sample was withdrawn, diluted, and plated to determine the initial spore count. The remainder of each heat-shocked DS medium culture was then heated at either 89 or 99°C for 1 min to 4 h or until the spore count reached 0 (depending on the individual isolate and the temperature being used). Sampling occurred every 30 s for 0–5 min, then every 60 s until 10 min, and 11–240 min every 30 min. At each time point, culture was mixed and a 100 μl time point sample was withdrawn and diluted (dilution range, 10^–2^ to 10^–7^). For vegetative cells, FTG (fluid thioglycolate) medium cultures grown for 24 h at 37°C were heated to 60°C for a time ranging from 1 min to 2 h. The time point samples were withdrawn as explained above. Finally, to determine the number of viable spores or cells present per milliliter (CFU/ml), the dilutions were plated on BHI. The logarithmic counts of viable spores and cells of every isolate were graphed against the heating time to determine the slope of the survival curve. The estimation of the D value was determined from this curve. P values were calculated using ANOVA analysis of variance (IBM SPSS Statistics for Macintosh, Version 27.0). Strains (955/85, CPI 57K-1, C269) did not produce spores with the methods used here.

### Maximum and Minimum Growth Temperatures

The growth temperatures of 30 *C. perfringens* strains were examined using the Gradiplate W10 incubator (Biodata Oy, Helsinki, Finland) placed in an anaerobic workstation (MK III, Don Whitley Scientific, Ltd., Shipley, United Kingdom) to determine the minimum and maximum growth temperatures (Korkeala et al., 1990; Derman et al., 2011). Briefly, strains were cultured in FTG at 37°C for 24 h. Strains were refreshed and grown at 37°C until turbidity OD_600_ reached 0.6–0.9. At least two biological replicates were cultured and analyzed. Cultures were diluted 1:10 in peptone water, and a sample of 25 microliters was transferred to a Gradiplate cuvette. Tested temperature gradients for minimum and maximum growth temperatures were 8–18°C and 47–57°C, respectively. Strains were incubated for 2 days for maximum temperatures and 21 days for minimum temperatures. Growth boundaries were determined by using a stereomicroscope, and the growth temperature threshold was determined as the boundary where dense bacterial growth was discontinued. Minimum and maximum growth temperatures were calculated as described by Korkeala et al. (1990) and Derman et al. (2011) using the formula T = T_low_ + d * g, where d is the distance (in millimeters) of the growth boundary to the measurement point of T _low_ and g is the temperature gradient.

## Results

### Phylogenetic Analysis Revealed Dispersal Patterns of *cpe*-Plasmids

Analyzed strains (*n* = 290) represented all five genetic lineages (I–V) and were isolated from a variety of animals as well as from humans and the environment (Supplementary Tables 1, 2). Here, lineages are numbered based on tree topology and are consistent with that used by Feng et al. (2020).

**FIGURE 1:**
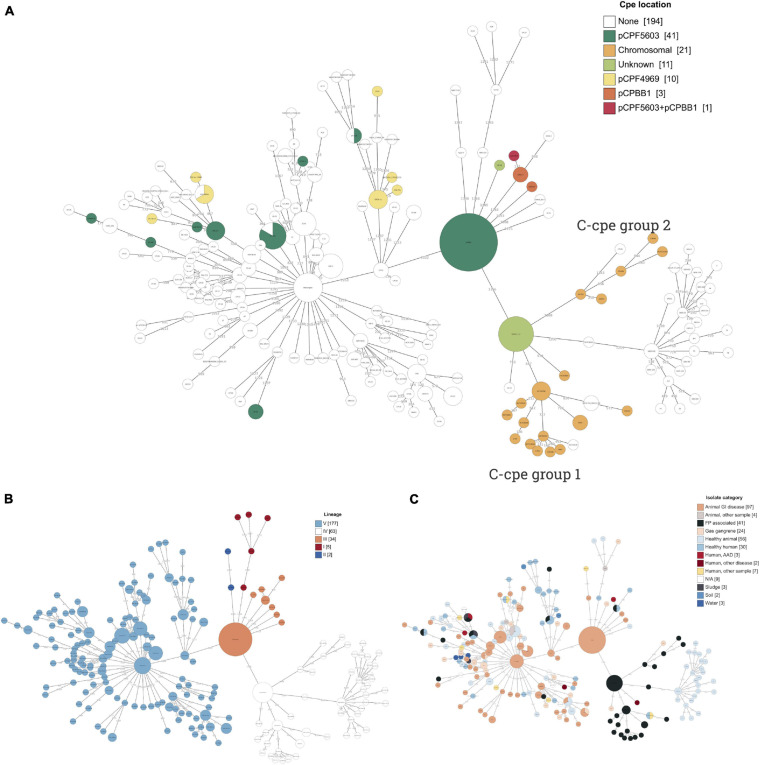
A minimal spanning tree of *Clostridium perfringens* strains based on cgMLST (scheme by Abdel-Glil, 2019) results annotated with **(A)** *cpe* location, **(B)** lineages I–V (Feng et al., 2020), and **(C)** isolate category based on their isolation source. Branches with a distance of less than 100 are collapsed. Plasmid pCPF5603 was widely distributed phylogenetically and present in two lineages, III and V. Contrastingly, pCPF4969 plasmids were present in two branches of lineage V. Lineage IV included 32 *cpe*-negative strains together with the food poisoning-related isolates with *cpe* gene inserted in their chromosome.

Three of the genetic lineages were well presented in sequenced strains: III (34 strains), IV (63 strains), and V (183 strains), and all *cpe*-carrying isolates belonged to these three lineages (Table 1). The majority of sequenced genomes (56.9%) were clinical isolates associated with veterinary (126/290) or human disease (39/290), and only 2.8% of genomes (eight, including the three environmental samples sequenced in this study) had been isolated from an environmental source such as soil, water, and sludge (Figure 1). Veterinary clinical isolates were scattered across all lineages, while all environmental isolates belonged to lineage V. Considering how common the AAD caused by *C. perfringens* is, it is surprising assemblies or closed genomes of AAD strains have not been published previously. In this study, three AAD isolates were included. The c-cpe genomes and one *cpe*-negative strain sequenced in this study belonged to lineage IV (n = 11), one AAD isolate to lineage III, and the rest to lineage V (*n* = 18). The minimal spanning tree of *C. perfringens* strains and their *cpe* type and isolation source based on cgMLST results are represented in Figure 1.

**TABLE 1:** Distribution of gastrointestinal (GI) disease isolates of human or animal origin and *cpe*-carrying isolates within lineages I–V.

Lineage	No. of genomes/isolates	% of isolates associated with GI disease	% of *cpe*-carrying genomes	*cpe* types (no. of genomes)
I	8	0	0	None (0)
II	2	50	0	None (0)
III	34	88	91	III/pCPPB1 (4), III/pCPF5603 (27)
IV	63	41	49	Chromosomally inserted *cpe* (21), unconfirmed *cpe* location on a draft genome (10)
V	183	44	14	V/pCPF4969 (10), V/pCPF5603 (15)

C-cpe strains (*n* = 31) all belonged to lineage IV. Phylogenetic analysis further revealed two subgroups of c-cpe strains (from now on referred to as the c-cpe group 1 and 2) and a closely related subgroup of *cpe*-negative isolates. In total, lineage IV included 32 (51%) *cpe*-negative isolates isolated from a variety of sources and geographic locations. These included 26 isolates from healthy swine and chicken (including the here sequenced European swine isolate 2a), stool of a healthy human (North America), two cases of gas gangrene in animals (Asia), and two food poisoning isolates (Europe). All c-cpe genomes had been isolated from food or stool samples linked to food poisonings in Europe.

Contrastingly, p-cpe strains (*n* = 52) belonged to lineages III and V and had been isolated from Europe, Asia, and North America. Figure 1A shows the dispersal patterns of *cpe*-carrying plasmids (pCPF5603, pCP4969, and pCPBB1) within genetic lineages. Plasmid pCPF5603 (*n* = 42) was the most widely distributed and present in both lineages III and V. Plasmid pCPF4969 (*n* = 10) was present in two branches of lineage V, which were phylogenetically related with each other’s and with lineage IV (Supplementary Figure 1). All strains carrying pCPPB1 plasmid (*n* = 4) with a variant of *cpe* toxin clustered together within lineage III (Table 1 and Figure 1A). The presence of toxin plasmids in other sequenced bacteria was looked up, but all three toxin plasmids were only found from *C. perfringens* genomes.

### Core Genome and Pangenome of *C. perfringens*

The core genome of the 290 strains consisted of, depending on the clustering method used, 1170–1660 coding sequences (CDSs), and the pangenome amounted to 14 306–23 148 CDSs (Table 2). The pangenome was open, and after 270 genomes had been added, the pangenome still expanded at a pace of approximately 10 new genes per added genome. The number of unique CDSs per genome varied from 133 (CP-21) to 0 (several genomes). The core genome presented 7–12% of the pangenome and 38–53% of the CDSs of an average genome (3120 CDSs).

**TABLE 2:** Core genome and pangenome estimations of *Clostridium perfringens.*

Study	No. of input strains (subgroup)	Pangenome Pipeline and chosen parameters	No. of core CDSs per core threshold Present in% of genomes			No. of CDSs in the pangenome	The proportion (%) of core genome (99%) of the pangenome and the average genome^b^
					
			100	99	95^a^		
This study	290	Panaroo, strict	1034	1660	2059	14306	11.6, 53.2
This study	290	Roary, 95% identity, split paralogs	231	590	1078	23148	2.5, 18.9
This study	283^c^	Roary, 95% identity, split paralogs	696	1170	1581	16875	6.9, 37.5
This study	86 (*cpe*-carrying strains)	Roary, 95% identity, split paralogs	1577	1577	1797	7835	20.1, 50.7
This study	86 (*cpe*-carrying strains)	Panaroo, strict	1941	1941	2101	7721	25.1, 63.2
This study	63 (lineage IV strains)	Panaroo, strict	1840	1840	2090	5917	31.0, 65.4
Kiu et al., 2019	110 (human enteric isolates)	Roary, 90%, don’t split paralogs	N/A	1965	N/A	∼6300	31.2, 63.2
Kiu et al., 2017	56	Roary, 95% identity, split paralogs	1470	1470	N/A	11667	12.6, 47.3
Abdel-Glil, 2019	76	Roary, 90%, split paralogs	N/A	N/A	2057	10098	20.3, 66.1^d^
Feng et al., 2020	173	Roary, parameters not specified	N/A	N/A	1020	26954	3.8, 32.8^d^

*^a^To enable comparison with previous results the core genome present in 95% of strains is given. Due to the concerns explained in the text, these should not be considered biologically relevant*

*^b^The average CDSs of analyzed genomes: 3120 CDSs for 290 strains, 3,071 for cpe-carrying strains, and 2814 for lineage IV strains.*

*^c^Confounding strains BER-NE33, K473, T3381, W1319, PC5, PBD1, and PBS5 were excluded from the analysis of 283 strains.*

*^d^95% core threshold was used instead of 99%.*

**FIGURE 2:**
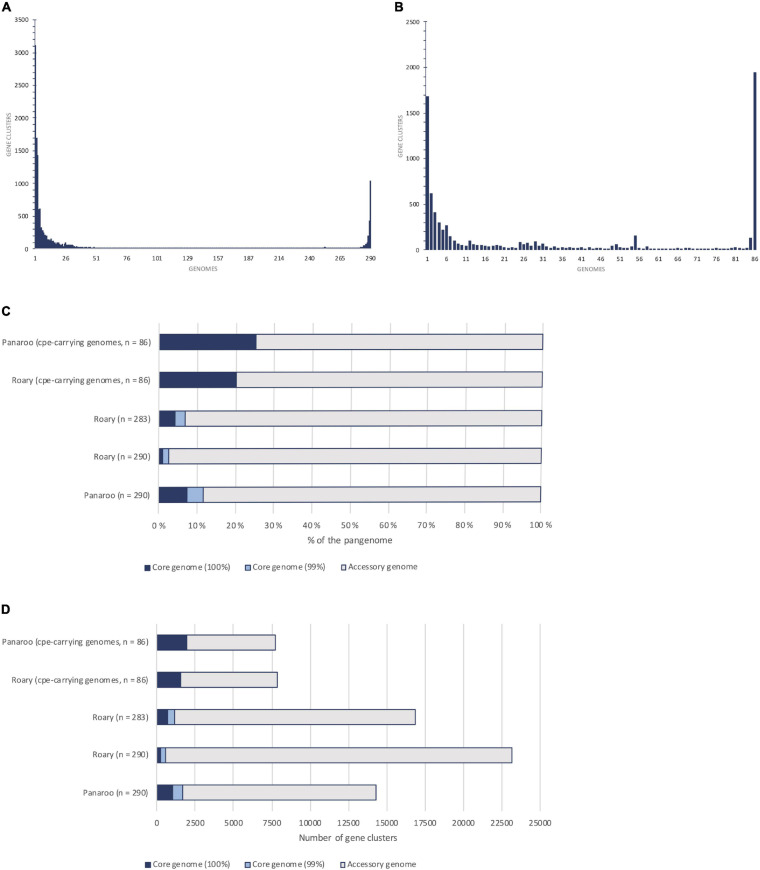
Pangenome and core genome comparison of *Clostridium perfringens* using Roary and Panaroo pipelines. **(A,B)** show the frequency of gene clusters within the pangenome, i.e., how many genomes were the gene clusters present in. **(A)** shows the gene cluster size distribution within the pangenome of 290 strains and **(B)** the gene distribution within the pangenome of 86 *cpe*-carrying isolates. Both **(A,B)** are based on Panaroo results. Chart **(C,D)** visualize the proportion of the predicted core genome to the accessory genome in different analyses based on the percentage **(C)** and the actual number of genes **(D)**.

The pangenomes of *cpe*-carrying strains (*n* = 86) and 290 strains were compared (Figure 2 and Table 2). For the *cpe*-carrying strains, the core genome covered 51–62% (1577-1941/3120) of the average genome CDSs and the pangenome amounted to 7721 CDSs. The core genome of *cpe*-carrying genomes was 17% (core genome present in 99% of strains) or 88% (core genome present in 100% of strains) larger compared to the core genome of all strains. The accessory genome of analyzed *cpe*-carrying genomes was also remarkably constrained (5780 CDSs) compared to that of all strains (12 646 CDSs). The most frequent gene cluster of the *cpe*-carrying genomes was the core genome (25%) (Figure 2).

Most of the genes (94%, 1044/1114) associated with *cpe*-carrying (*p* < 0.001, Benjamini-Hochberg adjusted) represented the accessory genome rather than shared characteristics between all *cpe-*carrying strains. Functional comparison of core genomes of 290 strains and *cpe*-carrying strains still revealed that the latter group shared more genes in 18 out of 24 clusters of orthologous groups (COGs). Especially genes belonging to COGs linked with amino acid metabolism and transport (*n* = 12), transcription (*n* = 18), signal transduction (*n* = 10), and unknown (*n* = 38) functions were more conserved in the core genome of *cpe-*carrying strains than the core genome of all *C. perfringens* strains. Operons related to riboflavin biosynthesis and sucrose metabolism, and three genes associated with drug resistance, bacitracin resistance gene *uppP* (CPR_1021), *mepA* (CPR_1218), and vancomycin resistance gene *yoaR* (CPR_1209), were fixed within the core genome of *cpe*-carrying strains, but not within the core genome (99%) of all *C. perfringens* strains.

The Panaroo pipeline produced a larger core genome with a smaller pangenome compared to the Roary pipeline in all settings (Figure 2 and Table 2). Based on manual curation of results, the presence of split CDSs or significantly truncated alleles generated most of this difference. Based on the conserved synteny the Panaroo clustered the broken sequences together with the conserved alleles, resulting in higher conservation counts for genes. In the clustering step for the Roary pipeline, the highly identical split CDSs fractionated the entire protein cluster to two or more separate clusters and consequently dropped these outside both the core genome of 99% strains as well as the soft-core present in 95% strains. Due to this, Roary did not return a reliable count of soft-core genes (present in 95% of genomes) for *C. perfringens* and stringent 99% or 100% core gene estimations were considered instead of 95% results (Table 2). Seven of the genomes had a notably high number of split reading frames/truncated alleles, and once these were removed from the analysis, the Roary pipeline produced a 98% larger core genome (83 CDSs per removed genome) (Figure 2 and Table 2).

### Conserved *cpe* Gene and a *C. perfringens* Strain With Two Plasmidial Copies of *cpe*

All but one of the strains (82/83) with a 319 aa *cpe* carried an identical copy of the enterotoxin gene either chromosomally or on a pCPF5603 or pCPF4969 plasmid. A single nucleotide mutation or sequencing error resulting in one amino acid change was observed in a previously sequenced strain (JFP981). Additionally, in all assembled c-cpe genomes (*n* = 21), the *cpe* gene was inserted between uracil-xanthine permease and *nadC* genes together with mobile elements, a transposase, and a hypothetical protein (75 aa) (Brynestad et al., 1997). Our study revealed no deletion scars of the *cpe-*carrying mobile element in the corresponding intergenic region of *cpe*-negative lineage IV strains.

In addition to 83 genomes carrying the 319 aa *cpe* gene, 5 genomes carried a 325 aa variant of the *cpe* gene. Noteworthily, one sequenced strain (AAD1903) contained both the 319 aa *cpe* gene and a 325 aa variant of the *cpe* gene. The 319 aa *cpe* was harbored on a pCPF5603 plasmid and the variant *cpe* on a pCPPB1 plasmid. Genetic regions surrounding the enterotoxin genes on plasmids pCPF5603 and pCPPB1 have been previously described (Miyamoto et al., 2011; Li et al., 2013) and are not repeated here. In addition to AAD1903, strains Q061.2 (pCPPB1), CP-09, a508.17 (pCPPB1), and a515.17 (pCPPB1) also harbored a 325 aa variant of *cpe* but did not carry the 319 aa *cpe* gene.

All five strains with the variant *cpe* clustered together in phylogenetic analysis (Figure 1A) and belonged to lineage III. Lineage III included 27 veterinary clinical isolates, mainly from necrotizing enteritis cases from foals and dogs from North America (*n* = 24), in addition to six other isolates (China, Great Britain, Finland, and France). Lineage III strains had larger genomes (*p*-value <0.001, ANOVA), lower GC% (*p*-value <0.001, ANOVA), and more CDSs (*p*-value <0.01, ANOVA) compared to lineages IV and V. All lineage III strains carried two copies of perfringolysin (PfoA). This variant of *pfoA* has an 85.7% nucleotide identity to the typical *pfoA* (Abdel-Glil et al., 2021).

### Lineage IV Showed Signs of Reductive Evolution, and the Strains Represented Two Distinct Gene Profiles

Lineage IV strains (*n* = 63) had a smaller genome (3.04 Mb, *p*-value <0.001, ANOVA) with fewer coding sequences (2814 ± 160 genes, *p*-value <0.001, ANOVA) than other lineages (*n* = 227, 3.43 Mb, 3204 ± 305 CDSs). Operons and genes absent from lineage IV were common in other lineages, suggesting that the ancestors of c-cpe strains carried these genetic characteristics and the genes have been lost on the course of evolution. Mobile genetic elements were more prevalent in lineage IV as annotation of 30 here sequenced strains revealed that lineage IV strains carried on average 99 mobile elements against the 27 found in p-cpe and related strains (p < 0.001, ANOVA), 172 repeat elements against 87 (*p* < 0.001, ANOVA), and 17 transposon sequences against 6 (*p* < 0.001, ANOVA) (Supplementary Table 3).

**FIGURE 3:**
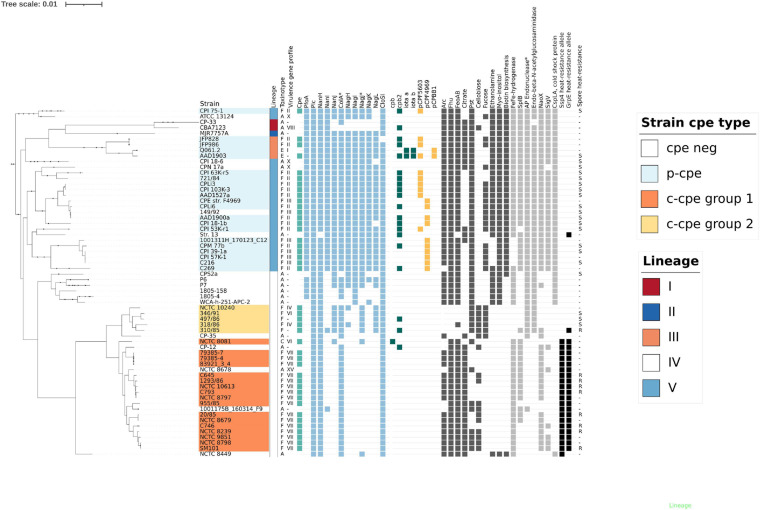
Lineages (I–V) (Feng et al., 2020), toxinotype **(A–F)** (Rood et al., 2018), virulence gene profile (I–XV) (Abdelrahim et al., 2019), together with selected genetic features (cpe, chromosomal, and plasmid-mediated virulence genes, toxin plasmids, operons, and genes differing between c-cpe strains) are visualized here to demonstrate the results of comparative genome analysis. The maximum-likelihood phylogenetic tree is based on the core genome of 290 strains, and sequenced 30 *Clostridium perfringens* genomes are shown together with representatives from each lineage. Squares signify the presence of operons or genes in each strain. The spore heat-resistance phenotype based on our results is indicated: “R” signifies heat-resistant phenotype (D_89°C_ over 100 min), “S” heat-sensitive phenotype (D_89°C_ 10 min or less), and “-” that spore heat-resistance phenotype for the strain is unknown. Arc = arginine deiminase pathway, Fhu = ferrichrome uptake system, FeoAB = iron uptake system, Citrate = citrate metabolism, Pst = phosphate ABC-transporter, Cellobiose = cellobiose metabolism operon, Fucose = Fucose metabolism operon, Ethanolamine = ethanolamine utilization operon, Myo-inositol = myo-inositol utilization operon, Biotin biosynthesis. **Genes** (light gray): SplB – spore photoproduct lyase, NaoX – CoA-disulfide reductase, SigV – sigma V. **Alleles and SNPs** (black): Ssp4 heat resistance allele = gene allele with Asp at residue 36 and Asn at residue 72 (J. Li and McClane, 2008), GrpE allele (CPF_0240) putatively associated with spore heat resistance (CPF_0240) with hydrophobic valine (V) at residue 37. Plasmid-mediated toxin genes *netB*, *etx*, *tpeL*, or *lam* were not present in any genomes presented here and have been omitted from this figure. * = Some genomes carried divergent ortholog, gene considered absent.

Comparative genome analysis showed that c-cpe strains carried fewer virulence genes than other strains. Within lineage V and III, the chromosomal virulence gene profiles were similar (Figure 3), but two groups of c-cpe strains (c-cpe group 1 and 2) had distinguishable gene profiles different from other lineages and the other c-cpe group. C-cpe group 2 encoded a more diverse virulence gene profile than c-cpe group 1 (Figure 3). Sialidase gene *nanJ*, μ-toxin homolog *nagJ*, glycoside hydrolase endo-β-N-acetylglucosaminidase, and hyaluronidase *nagL* are present in c-cpe group 2 but absent from c-cpe group 1. These genes were located chromosomally, and the genetic context around these genes was otherwise conserved in c-cpe and p-cpe strains, suggesting these genes had been deleted from c-cpe group 1.

In addition to differences in known virulence factors, c-cpe group 1 and 2 genomes were distinguishable by several genes and operons with predicted functions. The comparison revealed that c-cpe group 2 had lost, amongst others, arginine deiminase pathway operon, arsenic resistance operon, citrate metabolism operon, a paralog of iron uptake system FeoAB, iron transporting operon Fhu, and one homolog of [FeFe]-hydrogenase (Figure 3). C-cpe group 1 strains, on the other hand, had lost the above-mentioned virulence genes and also enzymes, fucose utilization operon, and carried a broken version of DNA repair mechanism (AP endonuclease family 2 protein) (Figure 3).

Comparative genome analysis by Lahti et al. (2012) revealed that c-cpe strains do not carry myo-inositol and ethanolamine utilization operon and also lack an operon for biotin synthesis. Our results add to this list a cold shock protein (CspLA, CPF_1452) and sigma V (SigV, CPF_0539) factor. SigV was absent from 25/31 c-cpe strains and 100% conserved in p-cpe strains (52/52). CspLA was conserved in 100% of p-cpe strains but absent from all c-cpe strains. Both c-cpe group 1 and group 2 carried very few phylogroup-specific accessory genes, 30 and 0, respectively. Genes unique to, and conserved within, c-cpe strains included 106 coding sequences, of which 26 were mobile elements or transposases, 49 hypothetical proteins, and 12 broken or truncated copies of genes (Supplementary Table 4). The most notable set of specific genes for lineage IV was the cellobiose utilization operon carried by 30% (19/63) of lineage IV strains and but absent from all but one lineage V and III strains. Cellobiose operon was flanked by phage and transposon-related genetic elements.

### Group of Chromosomal *cpe*-Carrying Strains in Lineage IV Produced Heat-Sensitive Spores

**FIGURE 4:**
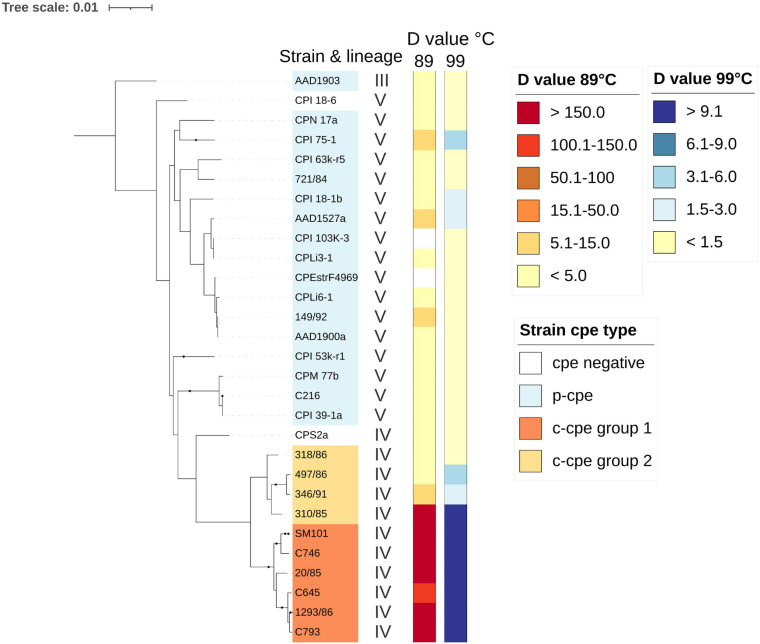
Spore heat-resistance results of *Clostridium perfringens* strains as D_89°C_ and D_99°C_ values. Strains from lineages III, V, and IV were assayed. Groups of chromosomal *cpe*-carrying strains produced heat-sensitive spores (c-cpe group 2) and were phylogenetically distinct from other c-cpe strains (c-cpe group 1). Measured D values (min) for *C. perfringens* strains are shown here against a maximum-likelihood phylogenetic tree of 29 *C. perfringens* strains (27 strains sequenced and heat resistance tested in this study and two reference strains: SM101 and CPE str. F4969). For SM101 and CPE str. F4969, D values were cited from previous publications (Li and McClane, 2008; Sarker et al., 2000). Strain 310/85 was an outlier in c-cpe group 2 producing distinctly heat resistant spores (D_89°C_ value 204 min).

To characterize the phenotypes of two different c-cpe gene profiles (Figure 3), the sequenced strains were assayed for heat resistance properties of their spores. C-cpe group 1 strains produced highly heat resistant spores with D_89°C_ values of over 100 min (average 226 min) and D_99°C_ values with an average of 36 min (Figure 4). C-cpe group 2 strains produced relatively heat-sensitive spores with D_89°C_ values of 3–9 min (average without strain 310/85 5.5 min) and D_99°C_ values with an average of 2.7 min. Strain 310/85 was an outlier in c-cpe group 2 producing distinctly heat resistant spores (D_89°C_ value 204 min). One representative of cpe negative group 3 strains was assayed and produced heat-sensitive spores. P-cpe strains and cpe-negative strains from lineage V and III produced spores with D_89°C_ values below 8 min (average 2.8 min) (Figure 4). Calculated D values of strains are available in Supplementary Table 6.

### Genes Associated With the Spore Heat-Resistance Phenotype

All c-cpe group 1 genomes carried the known heat resistance allele of Ssp4 (Asp at residue 36, Asn at residue 72), and all tested c-cpe group 1 strains produced heat-resistant spores. None of the tested strains that produced heat-sensitive spores carried the heat resistance allele of Ssp4 in their genome (Figure 3). However, one c-cpe group 2 strain (310/85) produced highly heat-resistant spores (D_89°C_ value 204 min) despite carrying the heat sensitivity associated allele (Gly instead of Asp, Lys instead of Asn) of Ssp4. It is, therefore, possible for *C. perfringens* to produce extremely heat-resistant spores without the heat resistance allele of Ssp4.

Differences between heat-sensitive and heat-resistant c-cpe strains were further analyzed. Orthologs that differed the most between heat sensitive and heat resistant c-cpe strains are summarized in Supplementary Table 7. Most of the sporulation-associated genes were highly conserved between strains, and the most divergent sporulation genes between heat sensitive and heat resistant c-cpe strains are listed in Supplementary Table 8. HGT elements were not associated with heat resistance. These comparisons revealed a cluster of 5 genes present in heat resistant strains (CPR_0713-0717) but absent from heat-sensitive c-cpe strains. Genes in this cluster encode for CoA-disulfide reductase NaoX and related permease and putative transcriptional regulator (Figure 3). Additionally, heat-sensitive c-cpe strains had lost a copy of GraRS two-component system, and type IV pilus operon genes *pilW*, *pilA*, and *pulG* were divergent (identity below 80%) between heat sensitive and heat resistant c-cpe strains.

Single amino acid substitutions (SNP) between c-cpe group 1 and group 2 strains were present in many sporulation and heat resistance-related genes (*ssp1*, *ssp2*, *spp3*, *clpX*, *clpC*, *clpP*, *grpE*, *groESL*, ribosome heat shock protein, *dnaJ*, *clpB*, spore coat protein S, *cotS*, *cotJC*, sporulation protein B) in addition to *ssp4*. Observed SNPs between c-cpe group 1 and 2 strains were consistent with phylogeny and did not differentiate 310/85 from the other c-cpe group 2 strains (data not shown). One exception to this was an SNP within a heat shock protein *grpE* (CPF_0240) that had an alanine substituted with hydrophobic valine on position 37 in all strains producing heat resistant spores including outlier strain 310/85 (Figure 3).

### Heat Resistance, Minimum, and Maximum Growth Temperatures of Vegetative Cells

Phenotypes of vegetative cells were characterized for heat resistance and growth temperatures. D_60°C_ values for vegetative cells ranged from 1 to 3.8 min (average 1.3 min), with no significant differences in heat resistance between c-cpe strains and p-cpe strains, or between the two c-cpe groups (Supplementary Table 6). The observed minimum and maximum growth temperatures for the strains ranged from 13.1 to 17.7°C and 49.6 to 52.0°C (Supplementary Table 6), respectively. None of the observed differences between genetic lineages or strain Cpe types were statistically significant.

## Discussion

In this study, we performed phenotypic, phylogenetic, and genomic analysis of enteropathogenic *C. perfringens* and showed that plasmidial *cpe*-gene is present in strains with certain genetic backgrounds. We also identified a new group of c-cpe strains and provided insight into the reservoirs and origin of c-cpe strains.

All *C. perfringens* strains have been presumed to be able to acquire horizontally transferable enterotoxin gene and produce enterotoxin. Based on our results, the *cpe*-carrying isolates can be divided into three main groups with two subgroups each: the lineage V isolates carrying either pCPF4969 or pCPF5603 plasmids, the lineage III isolates carrying either pCPF5063 or pCPPB1, and the lineage IV isolates with c-cpe group 1 and group 2 isolates. Isolates carrying plasmids pCPF4969 and pCPPB1 were phylogenetically close to their kin. Additionally, the accessory genome within the *cpe*-carrying isolates was smaller and the core genome larger than that of all strains. These results suggest that not all *C. perfringens* strains are as likely or perhaps even able to carry enterotoxin. One possible explanation for the here observed genetic context of *cpe*-carrying could be a biased transfer rate of conjugative plasmids favoring the plasmid transfer to the kin. The bias of the transfer rate of plasmids toward related strains has been described within natural populations of *E. coli* (Dimitriu et al., 2019). Alternatively, the drug resistance genes and other genes fixed within the core genome of *cpe-*carrying isolates might reflect the capacities required for strains to flourish at the reservoirs where toxin plasmids are shared. Further studies on enterotoxin plasmids and their incompatibilities in *C. perfringens* are warranted to understand the dispersal of the *cpe* gene between different strains.

Interestingly, c-cpe group 1 and c-cpe group 2 included also *cpe*-negative isolates, and the sequence comparison revealed that all c-cpe genomes had the *cpe* gene inserted in the same genetic location. This supports the notion of the clonal nature of c-cpe strains and suggests that *cpe* insertion to chromosome precedes diversification of c-cpe group 1 and group 2 strains. The *cpe*-negative strains have either persisted together with *cpe*-positive strains or, at some point, lost the chromosomal *cpe* gene. Since chromosomal *cpe* insert is carried in *Tn5565* (Brynestad et al., 1997), the latter explanation seems more probable. Proof for deletion of a *cpe-*carrying mobile element was not observed in the region between uracil-xanthine permease and *nadC* genes in *cpe*-negative lineage IV strains, but this should be further researched. For now, we hypothesize *Tn5565* has been cleanly removed through circulation from chromosomes of *cpe*-negative c-cpe group 1 and group 2 strains.

Noteworthily, here sequenced antibiotic-associated diarrhea strain AAD1903 carried two copies of plasmid-mediated c*pe* gene, one the well-known *cpe* gene of 319 aa sequence length (in pCPF5603 plasmid) and the other a 325aa variant (in pCPPB1 plasmid). The *cpe* variant in pCPPB1 plasmid is expressed (Miyamoto et al., 2011), but the clinical relevance of this variant *cpe* gene remains unknown. The presence of both versions of *cpe* in strain AAD1903 suggests these variants of enterotoxin might have different biological roles. To our understanding, this is the first time a single *C. perfringens* strain has been reported to carry two copies of the enterotoxin gene and two different enterotoxin plasmids. As all pCPPB1 plasmids were sequenced from isolates belonging to double *pfoA*-carrying lineage III, there seems to be potential within lineage III for the emergence of other double *cpe*, double *pfoA* strains similar to the strain AAD1903 described here.

*C. perfringens* genomes share a conserved genetic backbone, and more than half of the genes of an average genome are present in >95% of other strains. Two different methods for core genome and pangenome estimation were used in this study to probe the pangenome of this microbe. Pseudogenes and truncated alleles seem to be a typical mode of reductive evolution for *C. perfringens*, and the presence of broken CDSs creates issues when clustering-based algorithms are used to identify orthologs. Our results show that the exclusion of confounding genomes dramatically improved the pangenome prediction results. The Panaroo pipeline analyzing synteny handled broken CDSs better in the presence of confounding strains but risked the overestimation of functional core genome as pseudogenes and significantly truncated alleles were predicted as present. Despite the emergence of pangenome pipelines such as Roary, no standardized exclusion methods for poor-quality genomes are available. Here, we utilized genome sequence quality (Parrello et al., 2019) and frequency of truncated alleles in the cgMLST schema as inclusion criteria. In particular, truncated allele counts were useful in detecting genomes with a high frequency of broken reading frames (Supplementary Table 10).

The phylogenetic analysis here revealed that swine and chicken farm isolates sequenced by Li et al. (2021) are closely related with c-cpe strains and with a swine isolate sequenced here. Previously, c-cpe strains have been isolated solely from food and food poisoning related human samples, and their reservoirs and origin have been unknown. These findings are not yet directly indicative of the c-cpe reservoirs, but it is clear that the *cpe*-negative swine and chicken commensal strains are the closest known relatives of c-cpe strains. *Cpe*-negative members of lineage IV have not gathered much attention previously, and they have been rarely reported. However, the sequencing results of Li et al. (2021) suggest that lineage IV *cpe*-negative strains colonize the GI tract of domestic animals and are not rare, but rather underrepresented as they have been classified as type A strains. Sequencing of type A strains will likely help to uncover the reservoirs of c-cpe strains. Our results also show that p-cpe strains carrying pCPF4969 plasmid are phylogenetically closest to lineage IV (Supplementary Figure 1). Particularly, a group of lineage V strains mainly isolated from healthy humans (11/15), and carrying pCPF4969 (6/15), and having a smaller genome compared to other lineage V strains (*p* < 0.05, ANOVA) were phylogenetically close, suggesting that lineage IV and these putative members of human commensal GI population share ancestry.

Since the diversification from other lineages, lineage IV has undergone reductive evolution and niche adaptation. Supporting this observation, the c-cpe genomes have smaller genomes, fewer CDSs, few lineage-specific CDSs, and more HGT material compared to the other strains. Our results show that evolution within c-cpe strains has taken at least two directions and the two groups of c-cpe strains described here are likely adapted to different niches. C-cpe group 1 strains, but not group 2 strains, carry operons or genes linked to survival in harsh conditions and autolytic lifestyle (Arc operon, [FeFe]-hydrogenase, arsenic resistance/detoxification operon), competitive iron uptake (Fhu operon, FeoAB), and growth on citrate (Cit operon). An abundance of citrate is known to inhibit iron uptake through normal transport and instead, high-affinity siderophores such as ferrichrome (Fhu operon) are used (Pollack et al., 1970). In general, operons such as Fhu, Cit, and Arc are associated with acidophilic lifestyle, and paralogs of [FeFe]-hydrogenases are known to provide an advantage in changing pH conditions. C-cpe group 1 strains seem to be, compared to c-cpe group 2, better equipped for changing pH and sometimes acidic, high-temperature environments where iron uptake is competitive, and citrate utilization is beneficial. Previously, the reservoir for c-cpe group 1 strains has been proposed to be compost or sludge by Lahti et al. (2012). Both sludge and compost are acidic, prone to pH and temperature changes, abundant in citrate and iron sparse fitting to the profile described here. On the other hand, the loss of the aforementioned genes and operons makes it easy to speculate that pH changes and acidic stress are not characteristic of the adapted niche of c-cpe group 2.

Both c-cpe groups have lost virulence genes and genes common in intestinal bacteria and important for host colonization (Stahl et al., 2011; Becerra et al., 2015). C-cpe group 2 has, compared to c-cpe group 1, retained some genetic traits that likely provide a competitive advantage for host colonization and commensal lifestyle (fucose utilization operon, hyaluronidases *nagJ, nagL*, and sialidase *nanJ*). Adaptation to intestinal colonization is one possible explanation why the arginine deiminase pathway (Arc operon), despite its many uses in stressful conditions, has been deleted from c-cpe group 2 strains. Similar deletion of Arc operon has been suggested to be a trade-off between adaptation to urinary tract compared to the intestine in ESBL *E. coli*; Arc operon was beneficial for strains in the urinary tract, but a burden for mouse intestine colonization (Billard-Pomares et al., 2019). C-cpe group 2 genomes suggest adaptation to a less acidic environment, where the improved acquisition of anaerobic iron does not provide a competitive advantage. Their more diverse virulence gene profile is likely beneficial for commensal life and host colonization and group 2 strains may have retained a niche within the gastrointestinal tract of yet unknown host reservoir(s).

One possible big theme for reductive evolution within c-cpe strains could be an adaptation to planktonic growth. Soncini et al. (2020) have studied the gene expression of *C. perfringens* str. 13 growing on a plate, and many of the identified differentially expressed genes have been deleted from c-cpe strains. This means that the transcription of c-cpe strains growing on a plate differs greatly from those researched by Soncini et al. (2020). C-cpe strains also lack *pfoA*, important for biofilm formation (Vidal et al., 2015), and type IV pilus genes, important for quorum sensing and adhesion, are highly divergent between p-cpe, c-cpe group 1, and c-cpe group 2 (Supplementary Table 7). We hypothesize that c-cpe group 1 strains are adapted to environmental liquid niches and prefer planktonic growth. The preferences for liquid versus plated growth between *C. perfringens* strains should be further studied.

We studied heat-resistance and growth temperatures of *cpe*-carrying isolates and concluded that only c-cpe group 1 produced heat-resistant spores, while c-cpe group 2 and p-cpe spores are heat-sensitive. C-cpe strains have been characterized as heat resistant and clonal in previous studies, and to our understanding, this is the first time two separate groups of c-cpe strains with different virulence and functional characteristics have been described.

We also report one c-cpe strain to produce extremely heat-resistant spores without the presence of heat resistance associated allele of Ssp4. Li and McClane have previously shown that a single amino acid substitution in small acid-soluble spore protein beta or Ssp4 significantly affects spore heat resistance (Li and McClane, 2008; Li et al., 2009). Furthermore, genome analysis of 290 strains revealed that this heat resistance allele of Ssp4 was only found from c-cpe group 1 strains indicating this allele has most likely originated within this phylogroup. The capacity to produce highly heat-resistant spores seems to be characteristic and a putative adaptation for c-cpe group 1 strains. However, our results also indicate that other strains have the potential to acquire the capacity to produce highly heat-resistant spores and that heat resistance is not reliant on one allele but more likely to be affected by several genes.

The number of isolates in this study is not sufficient to deduce clear associations between heat-resistant phenotype and genotype within c-cpe strains. We identified two genes potentially associated with spore heat resistance in c-cpe strains: a certain allele of GrpE and the presence of CoA-disulfide reductase NaoX. GrpE in strains producing heat-resistant spores had a point mutation in the N-terminal part before helix structure. This N-terminal part has been proposed to act as a thermometer in *E. coli* (Gelinas et al., 2002), and alterations in this part may affect the thermal response of strain. CoA-disulfide reductase gene *naoX* has been deleted from c-cpe strains producing heat-sensitive spores. CoA is a common substance in dormant spores, and CoA-disulfide reductases have also been implied to have a role in the heat resistance of dormant spores (Setlow and Setlow, 1977; Hamilton et al., 2014).

To understand the ecology and epidemiology of this important pathogen, lineage-distinguishing typing method to identify closely related toxin-negative strains is needed. Lack of sequenced or lineage-typed environmental isolates compared to clinical isolates is likely contorting our view of *C. perfringens*, which after all is an environmental bacterium common in soil and water.

## Conclusion

Our study adds valuable genomic and phenotypic data to the publicly available collection of assembled and complete *C. perfringens* strains. The genomes include antibiotic-associated diarrhea isolates, *cpe*-positive environmental isolates, antibiotic-associated diarrhea isolate carrying double enterotoxin gene, and food poisoning isolates including strains representing the here described new subgroup of chromosomal *cpe*-carrying (c-cpe) isolates.

We established the signs of reductive evolution within lineage IV containing the c-cpe strains and elucidated their potential evolutive origin. C-cpe strains share ancestry with contemporary commensal strains isolated from swine and chicken. Comparative genome analysis revealed niche adaptation within the lineage of c-cpe strains. One subgroup produced heat-resistant spores and retained a genetic toolset suitable for survival in harsh environments. Meanwhile, the other subgroup produced heat-sensitive spores, lacked relevant operons for stress survival, but had conserved genes associated with host colonization and commensal lifestyle.

Focus on the rarely reported *cpe*-negative strains of lineage IV is likely to provide more insight into the ecology of c-cpe strains. Currently, the lack of genomic information about environmental isolates and the toxinotyping based on mobile elements is hindering research on ecology and reservoirs of *C. perfringens*.

Based on our results, it is apparent that spore heat resistance of *C. perfringens* strains is likely affected by multiple genes and the capacity to produce heat-resistant spores has developed primarily within one subgroup of chromosomal *cpe*-carrying strains.

## Data Availability Statement

The datasets presented in this study can be found in online repositories. The names of the repository/repositories and accession number(s) can be found in the article/Supplementary Material.

## Author Contributions

HK and KJ designed the study. KV performed the experiments. KJ performed the genome and data analysis and drafted the manuscript. KJ, PL, ML, and HK contributed to the data analysis and interpretation. KV, PL, RK-T, ML, and HK contributed to the manuscript revision. All authors have read and approved the final manuscript.

## Conflict of Interest

The authors declare that the research was conducted in the absence of any commercial or financial relationships that could be construed as a potential conflict of interest.

## Publisher’s Note

All claims expressed in this article are solely those of the authors and do not necessarily represent those of their affiliated organizations, or those of the publisher, the editors and the reviewers. Any product that may be evaluated in this article, or claim that may be made by its manufacturer, is not guaranteed or endorsed by the publisher.

## Footnotes

1
http://itol.embl.de/

## Supplementary Material

Supplementary Figure 1 Phylogenetic tree of 290 *C. perfringens* strains.

Supplementary Table 1 *Clostridium perfringens* strains sequenced ad reference genomes used in this study: the cpe genotype, isolation source, and isolation year.

Supplementary Table 2 Genomes included in the pangenome study.

Supplementary Table 3 Selected key factors of sequenced *Clostridium perfringens* genomes including plasmids and deposition keys.

Supplementary Table 4 Genes unique and conserved for c-cpe strains (bi-directional best hit orthologs).

Supplementary Table 5 Genes unique and conserved for c-cpe strain subgroups (bi-directional best hit orthologs).

Supplementary Table 6 Phenotyping results, heat-resistance assay results including D values for spores and vegetative cells, and minimum and maximum growth temperatures.

Supplementary Table 7 Genes divergent between in heat-resistant and heat-sensitive c-cpe strains.

Supplementary Table 8 Sporulation related genes (Xiao et al., 2015) divergent between c-cpe *Clostridium perfringens* strains and their identity against reference genes from *C. perfringens* SM101.

Supplementary Table 9 Presentation of genetic lineages within analyzed genomes, GC%, and genome size per lineage.

Supplementary Table 10 Number of truncated alleles per genome compared to cgMLST scheme.
