# Supplementary figures and images for: Comparative Genome Analysis and Spore Heat Resistance Assay Reveal a New Component to Population Structure and Genome Epidemiology Within Clostridium perfringens Enterotoxin-Carrying Isolates
Source: Front Microbiol. 2021 Sep 8;12:717176. doi: 10.3389/fmicb.2021.717176 (PMC8456093; doi:10.3389/fmicb.2021.717176)

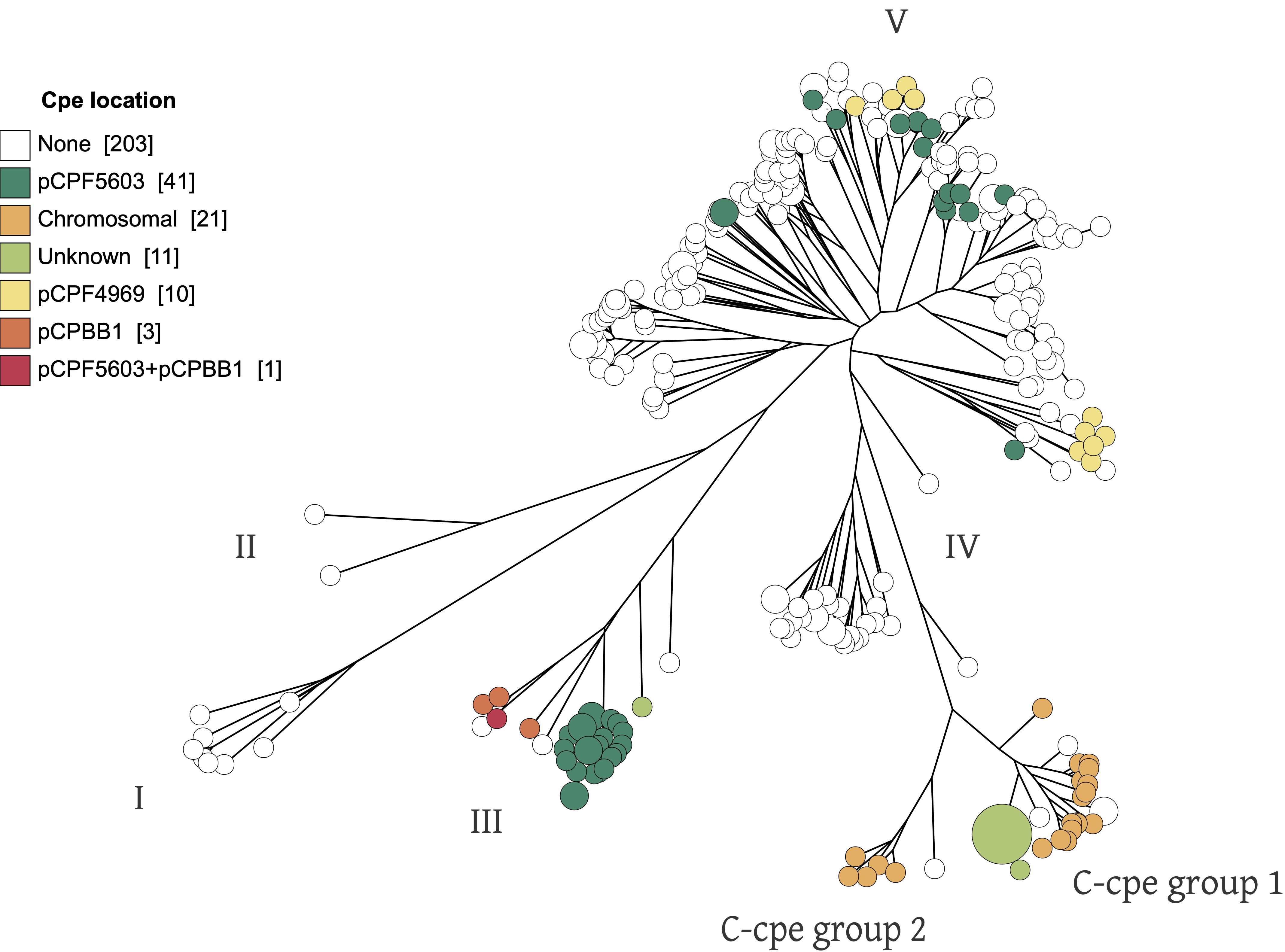

Supplement: Supplementary Figure 1 — Phylogenetic tree of 290 C. perfringens strains. [file Image_1.JPEG]
